# Effects of Social Attachment on Experimental Drug Use From Childhood to Adolescence: An 11-Year Prospective Cohort Study

**DOI:** 10.3389/fpubh.2022.818894

**Published:** 2022-03-29

**Authors:** Yi-Chen Chiang, Xian Li, Chun-Yang Lee, Chi-Chen Wu, Hsing-Yi Chang, Shuoxun Zhang

**Affiliations:** ^1^State Key Laboratory of Molecular Vaccinology and Molecular Diagnostics, School of Public Health, Xiamen University, Xiamen, China; ^2^School of International Business, Xiamen University Tan Kah Kee College, Zhangzhou, China; ^3^Institute of Population Health Sciences, National Health Research Institutes, Miaoli, Taiwan; ^4^Business School, Sichuan University, Chengdu, China

**Keywords:** experimental drug use, social attachment, cohort study, childhood, adolescence, survival analysis

## Abstract

**Background:**

Drug use among adolescents are still crucial issues that endanger their lifetime health. Evidence concerning the interpersonal-related factors influencing youngsters' experimental drug use behavior, especially from longitudinal and school-based prospective cohort studies, is insufficient. We aimed to describe the annual incidence rate and mean annual incidence rate of experimental drug use from childhood to adolescence by education stage, clarify the risk in childhood and examine the longitudinal relationship between social attachment factors and experimental drug use.

**Materials and Methods:**

The data were derived from the 1st to 11th wave of the longitudinal study. In total, 1,106 respondents aged 19–20-year-old were followed up for 11 years (from 9 to 10-year-old) in Taiwan. A survival analysis was used to analyze the time-invarying/time-dependent effects of social attachment factors on experimental drug use.

**Results:**

The mean annual incidence rate of experimental drug use from childhood to adolescence was 6.8‰. The incidence increased over time and was the highest in the first year of university (19.3‰). Boys were more likely to use drugs than girls. A low degree of self-perceived likeability in childhood was a risk factor influencing experimental drug use. On average, a low degree of parental supervision and a high degree of family conflict were both influential risk factors. According to the time-dependent models, a high degree of parental supervision, a high degree of family support and a low degree of family conflict in the current year can protect children and adolescents from drug use, whereas a sustained low degree of parental supervision and a high degree of family conflict may promote students' experimental drug use.

**Conclusion:**

Parents should be informed and educated to avoid family conflict during childhood, maintain consistent supervision of their children's behavior, provide adequate family support, and pay attention to their children's interpersonal relationships in school. Teachers should focus on the social attachment status of their students while considering their attachments to their families and peers.

## Introduction

Drug use is increasing worldwide, which can be manifested by the augmenting total number and proportion of drug users. In 2018, an estimated 269 million people worldwide used drugs at least once in the previous year, corresponding to 5.4% of the global population aged 15–64 years (1). The estimated number of any drug users in the past year globally rose from 210 million to 269 million during the period between 2009 and 2018; thus, the growth rate of drug consumption was more than a quarter (28%) (1). In 2018, an estimated 35.6 million people suffered from drug use disorders globally (2). There is a strong link between drug use disorders and psychiatric comorbidities, and these conditions often share common risk factors (3–6), such as family structure and functioning, family psychiatric and substance abuse history, traumatic events, peer relations and peer group characteristics (5). In addition, the COVID-19 pandemic has raised huge concerns for the mental health of an entire generation of children and young people (7), which may be related to the adoption of anti-epidemic measures such as lockdown, school closure, and social distancing. Mandatory social distancing policies have reduced the accessibility of drugs, and adolescents' drug use has decreased (8, 9); while subsequent small-scale unblocking might bring about experimental drug use among adolescents. Drug use have become a public health issue with a serious impact on people's development and social security. It is therefore necessary to further explore the prevalence and negative effects of drug use among children and adolescents.

### The Prevalence and Negative Effects of Drug Use From Childhood to Adolescence

Adolescence is an important transitional period during people's lifespan characterized by physical and psychological development. For some adolescents, adolescence is also a time of increased vulnerability to the initiation of drug use. In 2018, it was estimated that there were 13 million past-year users of any drug among students aged 15–16 years globally (10). According to studies in the United States, New Zealand and Australia, this rate increased to 17% among lifetime users who started using cannabis in adolescence (11). Based on the Monitoring the Future Survey (MTF) of America, 26.4, 40.8, and 51.8% of the respondents had tried an illicit drug in 8th grade, 10th grade, and 12th grade, respectively (12). A study conducted in Canada followed 4,885 adolescents throughout secondary school and found that 17.6% of these adolescents reported using illicit drugs in 7th grade (13). In a survey involving 2,974 Japanese junior and senior high school students, 3.8% reported using illicit drugs (14). A national survey of drug use in China found that among the 2.553 million reported drug users, 0.6% were under the age of 18 (15). A 3-year survey conducted in Taiwan indicated that 1.5% of the 2,375 students previously used drugs (16). This difference in the prevalence of drug use may be due to cultural differences or prevention policies. For example, students in Taiwan complete a series of drug prevention courses at various stages since elementary school. However, research focusing on the onset of drug use behavior among children and adolescents is insufficient, thus deserving further attention.

The long-term use of drugs can lead to physiological tolerance and psychological dependence, resulting in addiction. Adolescent drug use entails health risks (17). It is significantly associated with other leading causes of morbidity and mortality during adolescence, including depression, anxiety, unprotected sex, suicide attempts, and accidents (18–21). Drug use during adolescent was associated with their cognitive control and emotion regulation (22). The results of a nationally representative sample survey of 10th graders in the United States showed that polysubstance users reported elevated levels of somatic and depressive symptoms (23). Hence, this study further explored the influencing factors of drug use among children and adolescents.

### Theoretical Research Review

Humans depend on social relationships for survival and wellbeing throughout life (24). Social control theory indicates that “social attachment” is the earliest social connection in children. “Attachment” refers to the affective relationships that one has with other people (25). Social attachment has the basic attributes and functions of attachment, which focuses on the emotional characteristics and behavioral tendencies that individuals form with specific objects during their growth (26). Social attachment can be considered as a social bond, and this bond can be either secure or insecure (27, 28). A secure bond can be described as a balanced interaction in which the interactors are neither too distant nor too close (29). It provides a sense of wellbeing, intimacy, or security to interactors (30). Positive bonds to society deter adolescents from substance use (31).

Attachment theory, which is formulated by Bowlby (32–34), conceptualizes the tendency of individuals to build strong emotional bonds with specific others and understands varied forms of affective disturbance (e.g., anger, despair, and detachment). Attachment relationships are usually formed during infancy and tend to be relatively stable after adolescence (35). Hence, attachment relationships from childhood to adolescence are extremely important (32). As social attachments increase, participation in health protective behaviors increases (36). Notably, social attachments in childhood and adolescence are mainly derived from family and friends (37). Like social attachments, social learning theory emphasizes that cognition affecting behaviors stem from observing others (38, 39). For children and adolescents, the most influential role models are parents, peers, and siblings (40). Vicarious learning requires an identification process when the students match or identify themselves with the punishment/reward target so that they will subsequently have contiguous association of the sensory event. Their self-controlling responses can be modified and reinforced by models' self-evaluative and self-punitive reactions to deviation (41). During the identification process, the child/adolescent decides which actions should and should not be done based on the perceived consequences of the actions. In order to reinforce constructive parent-child/peer interaction and dynamic, students tend to enhance their positive behaviors and inhibit non-expected behaviors.

Family environment and atmosphere have an impact on the development and behavior of children and adolescents (42). Children may model parents' behaviors which have greater or lesser access to health-harming substances, receive specific encouragement or discouragement to participate in certain behaviors, and receive family support to attempt and alter behaviors (43). Research has documented that poor family interactions, low levels of family supervision, and parental use of punishment for discipline all increase the risk of future substance use (44–46). Similarly, peer influence was a significant factor on adolescents' participation in health-related behaviors such as substance abuse (47, 48) and drinking behavior (49). Youngsters who were popular and liked within their peer group were rated as more competent within their closest friendship (50). Students who lack supportive friendship network and have low adaptability to school are more likely to be exposed to drugs (36, 51, 52). In particular, perceived likeability impacts children's subsequent substance abuse and dependence (53). Therefore, the social attachment of this study includes four aspects: parental supervision, family support, family conflict and perceived likability. Clearly, social attachment is critical to children's and adolescents' healthy development. However, previous studies did not examine the impact of social attachment on children's or adolescents' substance use from long-term perspectives, especially in multiyear prospective cohort studies.

### The Present Study

The prevalence of drug use among children and adolescents reviewed in the above literature is primarily based on cross-sectional data, and it is difficult to see changes in drug use at different consecutive stages. Based on 11-year longitudinal data, the present study was meant to determine the new incidence of experimental drug use among students by year and education stage. According to the continuous tracking of periodic fluctuations in drug use among children and adolescents, we hypothesized that adolescents with weaker social connections were more likely to start drug use than their counterparts. This study further examined the impact of students' social attachment factors on experimental drug use by longitudinal cohort. Social attachment factors involved their parents and peers (in childhood and time-dependent contexts), i.e., parental supervision, family support, family conflict, and perceived likeability. Therefore, our objectives were to (a) describe the annual incidence rate and mean annual incidence rate of experimental drug use from childhood to adolescence by education stage; and (b) examine the longitudinal relationship between social attachment factors and experimental drug use behavior throughout the education duration, including early/average exposure and simultaneous/lag effects.

## Materials and Methods

### Participants and Survey Procedures

The data were derived from the 1st to 11th wave of the longitudinal study Child and Adolescent Behaviors in Long-term Evolution (CABLE), which was initiated in 2001 (54). There were 152 primary schools in urban area and 79 primary schools in rural area. There were only a few private primary schools in these two areas (10/152 in urban and 1/79 in rural). Differing from students attending public schools, those who attended private schools have relatively good family conditions. In addition, the overall teaching mode in private schools might differ from the compulsory education system of public schools. Furthermore, most students attended public schools. Thus, these private schools were excluded from the sample population in the cohort study after considering the comparability of the study subjects. Based on the number of first-grade students, the schools were divided into small (50–199 students), medium-sized (200–399 students) and large (more than 400 students) schools. Schools with fewer than 50 students were not included in the sample population due to insufficient numbers. Then, the schools were randomly selected to participate in the survey. To ensure that the numbers of children chosen from each type of school were approximately equal, six small schools, two medium-sized schools and one large school were selected from each location. Finally, eighteen public elementary schools from urban area and rural area were randomly selected. The school/parents/students were informed of the purpose of the cohort study, and written informed consent for the annual follow-up survey was obtained from the students' parents before the baseline survey. Since this article presents a secondary analysis, the data were released with deidentification to protect the privacy of the children. The study team did not intervene or inform the teachers/parents of the students' actual answers.

**Figure 1:**
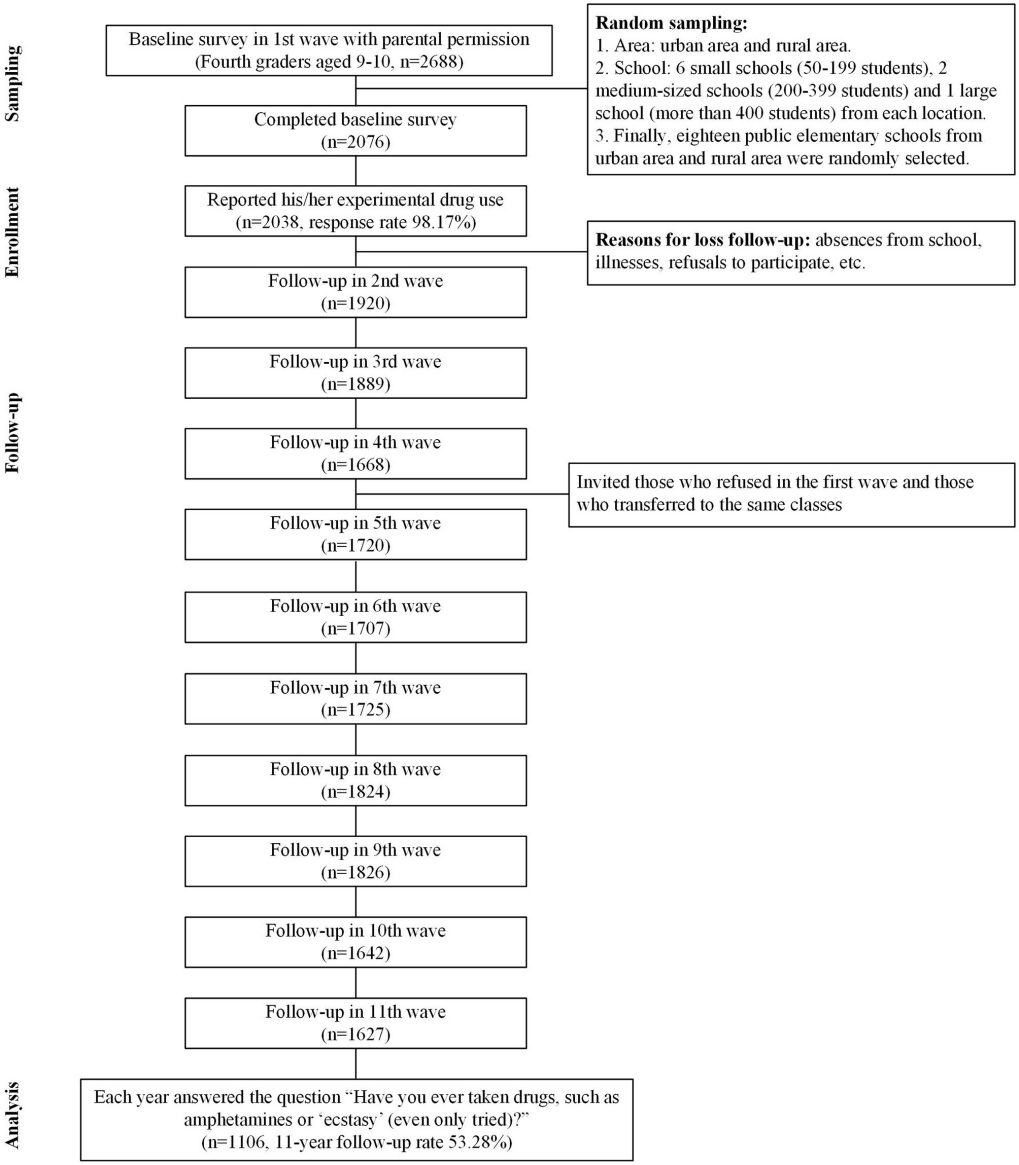
Flowchart.

All 3,612 fourth graders aged 9–10 years were selected. Among them, 2,125 students provided parental permission in 1st wave (58.83% agreed to participate). Moreover, 2,076 students completed their questionnaires (response rate = 97.69%). The annual losses to follow-ups were due to absences from school, illnesses, refusals to participate, etc. (annual response rate = ~78.37–98.17%, Figure 1). Eventually, in total, 1,106 respondents aged 19–20 years completed an annual survey (11-year follow-up rate = 53.28%). Fortunately, when comparing the distributions of the students' sex, parents' education levels and students' drug use behaviors between “the first-wave data” and “the 11-year follow-up data”, the results were non-significant. We assumed that cohort data were randomly missing. Further details of the sampling procedures of the cohort study have been previously described (55). Furthermore, participants completed a follow-up questionnaire in classes/schools before their senior high school years. Trained interviewers answered questions posed by the students, which verified that they understood each question. As the participants grew up during senior-high-school/college years, they were followed up face-to-face at home/school. Because the students were enrolled in different middle schools and universities after their elementary school years and because they could have different experiences, the clustering effect was expected to be at a minimal level.

### Measurements

#### Experimental Drug Use

Drug use refer to the use of substances controlled under the international drug control conventions for non-medical (56). Experimental drug use is defined as having tried a drug, which may develop into habitual drug use through enduring exposure to drugs, or may be reversed with timely intervention (57, 58). This variable was measured by asking the students “Have you ever taken drugs, such as amphetamines or “ecstasy” (even only tried)?”. Among the annual surveys, the response options differed depending on the age of the respondents: (a) The students in elementary school grade 4 or grade 5 responded using a five-point scale with “never” = 1, “not recently but in the past” = 2, “once or twice in the past month” = 3, “many times in the past month” = 4, and “every day in the past month” = 5, and (b) the students from grade 6 to grade 14 responded using a six-point scale with “never” = 1, “not in the past year but before” = 2, “not in the past month but before” = 3, “once or twice in the past month” = 4, “many times in the past month” = 5, and “every day in the past month” = 6. The time (school grade) of experimental drug use was used as an outcome variable in the survival analysis. Experimental drug use at baseline included students before the fourth grade. Therefore, these students were asked the exact year of the experimental drug use that occurred before grade 4.

#### Social Attachment

The independent variables included parental supervision, family support, family conflict, and perceived likeability. These four factors were measured consistently in most of the 11 years, for they can be used to test their long-term influence on the students' experimental drug use. The measurement and scoring of each item were conducted as follows.

*Parental supervision* was measured from grade 6 to grade 9 using a scale with three items, including “Does your mom or dad know what you do after school before returning home every day/who you usually go out with/what you do in your free time?”. These items were scored on a four-point scale with higher scores indicating higher levels of parental supervision.

*Family support* was measured annually in our 11-year data set by using six items, including “Does your mom or dad care for you when you are feeling unwell?” and “Does your mom or dad comfort you when you are feeling unhappy or sad?” These items were scored on a four-point scale with higher overall scores indicating higher levels of family support.

*Family conflict* was measured from grade 4 to grade 9 by using five items, including “In the past month, did your parents hit each other when they were fighting?”. These items were scored on a four-point scale with higher overall scores indicating higher levels of family conflict.

*Perceived likeability* was measured annually in our 11-year data set by using five items, including “In the past two weeks, were you afraid that your friends or other people don't like you/people are making fun of you”. These items were scored on a three-point scale with higher scores indicating that the respondent was more likely to perceive that they were not well-liked (i.e., low likeability).

These scales all demonstrated acceptable reliability with Cronbach's α values of 0.69–0.83 for parental supervision, Cronbach's α values of 0.80–0.92 for family support, and Cronbach's α values of 0.65–0.84 for perceived likeability. In the confirmatory factor analysis (CFA), parental supervision only includes three items, so degrees of freedom (df) = 0, which cannot provide the index of the goodness-of-fi; the factor loading values of the three items are 0.90–1.00 for parental supervision. The CFA results of family support showed: Adjusted Goodness of Fit Index (AGFI) = 0.99, Comparative Fit Index (CFI) = 0.99, Incremental Fit Index (IFI) = 0.99, Critical N (CN) = 809.45. The CFA results of perceived likeability showed: AGFI = 0.97, CFI = 0.94, IFI = 0.94, CN = 277.96. The above CFA results show that the scale is valid (59–61).

#### Control Variables

Although previous studies have suggested that students' drug use was associated with sex (62), subjective academic performance (63, 64), parents' education levels (65, 66), etc., these factors were not the focus of this study and were deemed as control variables.

### Statistical Analysis

Experimental drug use in the sample is described using frequencies and percentages. For the incidence of experimental drug use, the annual incidence rate refers to the number of newly reported experimental drug users in each year out of the total number of students who never used drugs up until the previous year, except for the annual incidence of experimental drug use in the first year, which represents the cumulative incidence (i.e., includes the frequency of drug use prior to grade 4). The independent variables are described using means and standard deviations. In this study, for those participants who answered more than half of the questions on a scale, we measured the individual average values of the remaining questions to replace the missing values (67, 68). Otherwise, these values were treated as completely missing. A Cox regression was used to examine the relationship between each variable and experimental drug use, which helped to figure out the causal relationship between the independent and dependent variables over time. We examined the proportional hazards assumption using the Schoenfeld test, and the results showed no violation of proportionality (Appendix 1). Thus, the proportional hazard model was used in this study.

To examine early/average exposure or the long-term effect, Cox proportional hazards models were created using the following two different approaches: (a) for the time-invarying covariates, model 1-1 assessed the hazards of early exposure in childhood, whereas model 1-2 assessed the hazards of the average exposure during the 11 years of follow-up, and (b) for the time-dependent covariates, model 2-1 examined the simultaneous effects, whereas model 2-2 examined the lag effects. Regarding those variables that were only assessed at early ages, we measured the average values. When considering only those variables that were measured in all years, we modeled these variables as time-dependent covariates. In addition, instead of replacing each missing value with a single value, a multiple imputation (MI) model replaced each missing value with a set of plausible values that represented the uncertainty of the correct value to impute (69, 70). Via comparisons between our analyzed data collected before and after the MI procedures (with the comparisons assessed via R software using the Amelia II strategy to replace the missing values), we found the results were similar (i.e., the differences in each HR value assessed before and after the MI were almost lower than 0.02). SAS software version 9.2 was employed for the statistical analysis.

## Results

### Sociodemographic Characteristics

As shown in Table 1, the respondents aged 9–10 years old were approximately half boys (51.4%) and half girls (48.6%). In total, 53.8% of the participants were from urban areas, and 46.2% of the participants were from rural areas.

**Table 1:** Characteristics of the sample of fourth graders (aged 9–10 years) in the baseline survey (*n* = 2,688).

**Characteristics**	***n***	**%**
**Sex**		
Boys	1,382	51.43
Girls	1,305	48.57
**Mother's/Father's highest level of education**		
Elementary school	29	1.10
Junior high school	164	6.20
Vocational school	762	28.79
Senior high school	201	7.59
Technical college	562	21.23
University	645	24.37
Graduate school	284	10.73
**Residential location**		
Urban	1,447	53.83
Rural	1,241	46.17

*All 2,688 students in the extracted schools were received parental permission and completed their questionnaire forms during the 11 years. One student didn't report his/her gender. Forty-one students didn't report their mother's/father's highest level of education*.

### Incidences of Experimental Drug Use From Childhood to Adolescence

**Figure 2:**
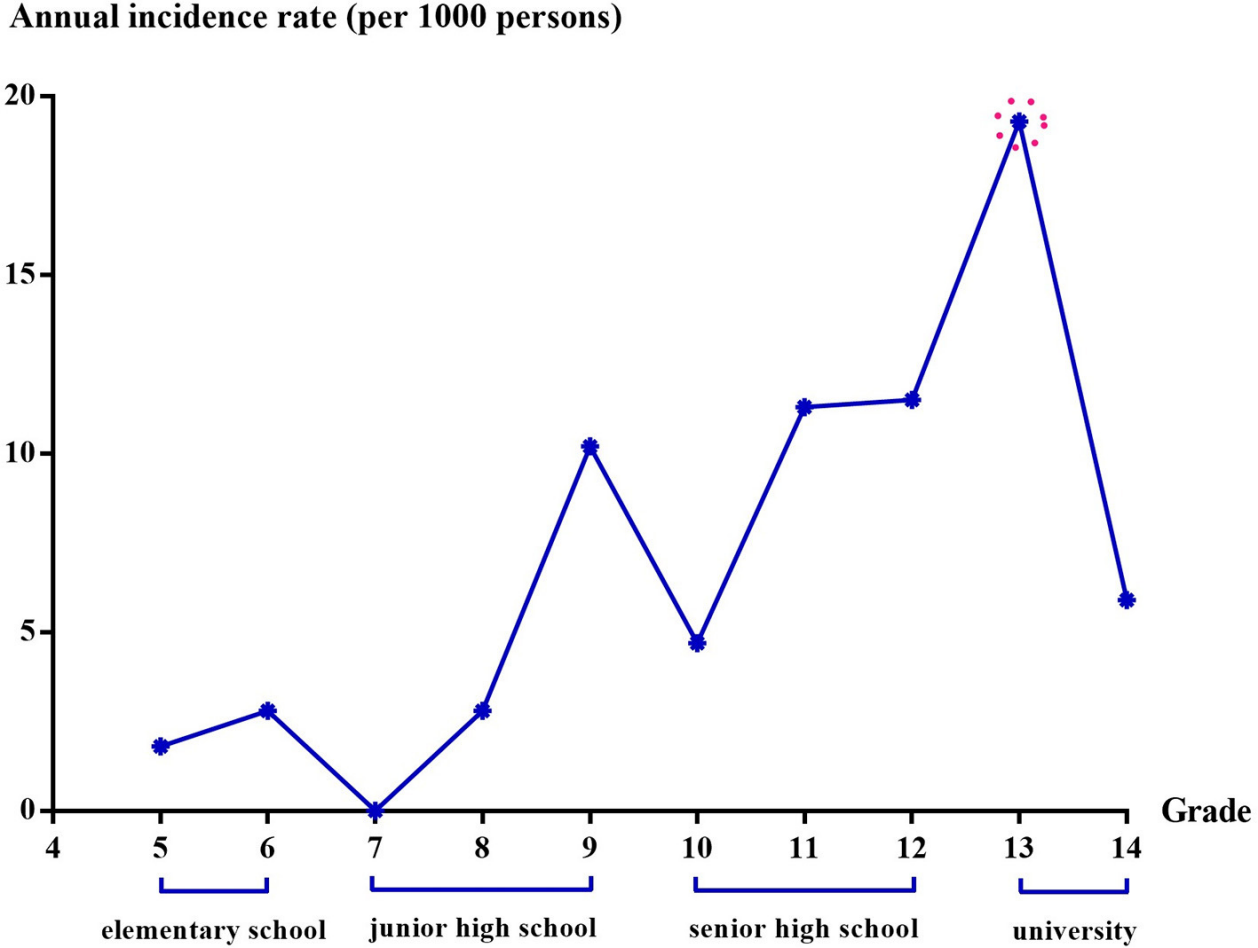
Annual incidence rate of experimental drug use.

As shown in Table 2, the rate of drug use in 1st wave (21.7 per 1,000 persons) represents a cumulative incidence and includes the frequency of using drugs prior to 4th grade aged 9–10 years old. The highest mean annual incidence rates from 5th grade (aged 10–11 years old) to 14th grade (aged 19–20 years old) were observed in 13th grade (aged 18–19 years old), was 19.3‰. This finding indicates that new cases of experimental drug use were the highest upon attending university. The mean annual incidence rates gradually increased by education stage. In addition, there were two peaks of students' experimental drug use: 9th grade aged 14–15 years old (the third year of junior high school) and 13th grade aged 18–19 years old (the first year of university) (Figure 2). Experimental drug use in senior high school and university was much higher than that in the other stages (Table 2).

**Table 2:** Frequency, annual incidence rate and mean stage incidence rate per 1,000 persons of experimental drug use among 11-year follow-up participants.

**Grade**	***n***	**Annual incidence rate (per 1,000 persons)**	**Education stage**	**Mean annual incidence rate by education stage (per 1,000 persons)**
Grade 4	24	21.7^a^	Elementary school	2.31^c^
Grade 5	2	1.8^b^		
Grade 6	3	2.8^b^		
Grade 7	0	0.0^b^	Junior high school	4.33^d^
Grade 8	3	2.8^b^		
Grade 9	11	10.2^b^		
Grade 10	5	4.7^b^	Senior high school	9.09^d^
Grade 11	12	11.3^b^		
Grade 12	12	11.5^b^		
Grade 13	20	19.3^b^	University	12.57^e^
Grade 14	6	5.9^b^		
Total	98	6.8^f^		

a *Annual incidence rate of experimental drug use in first year represents cumulative incidence that includes the frequency of using drugs prior to grade 4*.

b Annual incidence rate = (new reports of experimental drug use/total number of those reporting never using drugs up until the previous year) * 1,000‰.

c Mean annual incidence rate by education stage of experimental drug use in elementary school = (new reports of experimental drug use in grade 5 and grade 6/total number of those reporting never using drugs up until grade 4)/2 * 1,000‰.

d Mean annual incidence rate by education stage = (new reports of experimental drug use in this educational stage/total number of those reporting never using drugs up until the previous stage)/3 * 1,000‰.

e Mean annual incidence rate by education stage = (new reports of experimental drug use in this educational stage/total number of those reporting never using drugs up until the previous stage)/2 * 1,000‰.

f Mean annual incidence rate of drug use in the 11-year follow-up = (new reports of experimental drug use with 11 years of follow-up/the number of exposed people in this population during the same period)/number of years * 1,000‰ = [(98–24)/(1,106–24)]/10 * 1,000 ‰ = 6.8‰.

### The Hazards or Protection of Social Attachment Factors on Experimental Drug Use

As shown in Table 3, boys were significantly more likely to start using drugs during the follow-up than girls, and students living in urban areas were more likely to consume drugs than students living in rural areas. Model 1-1 revealed that a low perceived likeability in childhood was a risk factor influencing experimental drug use [hazard ratio (HR) = 1.15, 95% CI = 1.02–1.30]. Students with worse subjective academic performance (HR = 0.73, 95% CI = 0.51–1.04) in elementary school were marginally and significantly more likely to start using drugs. Besides, model 1-2 indicated that low parental supervision (HR = 0.83, 95% CI = 0.71–0.98) and high family conflict (HR = 1.24, 95% CI = 1.01–1.52) on average were both risk factors influencing experimental drug use.

**Table 3:** Relationship between social attachment factors at different time points and experimental drug use: survival analysis results.

**Variables**	**Time-invarying effects**		**Time-dependent effects**	
	**Cox PH model 1-1 (early exposure)**	**Cox PH model 1-2 (average exposure)**	**Cox PH model 2-1 (simultaneous effects)**	**Cox PH model 2-2 (lag effects)**
	**HR (95% CI)**	**HR (95% CI)**	**HR (95% CI)**	**HR (95% CI)**
**Control variables**				
Sex (boys/girls)	2.25 (1.18, 4.26)^*^	1.88 (1.09, 3.24)^*^	1.83 (1.07, 3.13)^*^	2.06 (1.17, 3.63)^*^
Subjective academic performance	0.73 (0.51, 1.04)^†^	1.07 (0.63, 1.80)	1.13 (0.68, 1.88)	1.10 (0.64, 1.87)
Parents' education level	0.94 (0.76, 1.17)	0.98 (0.81, 1.18)	0.95 (0.79, 1.15)	0.97 (0.81, 1.18)
Residential location (urban/rural)	1.96 (1.03, 3.73)^*^	1.70 (0.98, 2.94)^†^	1.74 (1.001, 3.01)^*^	1.85 (1.04, 3.30)^*^
**Social attachment factors**				
Parental supervision	1.00 (0.89, 1.14)	0.83 (0.71, 0.98)^*^	0.85 (0.74, 0.99)^*^	0.77 (0.66, 0.89)^***^
Family support	0.97 (0.90, 1.05)	0.93 (0.85, 1.03)	0.91 (0.86, 0.97)^**^	1.00 (0.98, 1.01)
Family conflict	1.16 (0.99, 1.37)^†^	1.24 (1.01, 1.52)^*^	1.26 (1.03, 1.53)^*^	1.23 (1.001, 1.52)^*^
Low perceived likeability	1.15 (1.02, 1.30)^*^	1.04 (0.87, 1.25)	1.05 (0.94, 1.16)	1.01 (0.97, 1.04)

*In model 1-1 (early exposure in childhood), students' subjective academic performance and parental supervision were measured for the first time in 3rd wave, and other variables were measured in 1st wave. In model 1-2 (average exposure during 11 years of follow-up), students' sex and residential location were measured in 1st wave, parents' education level was check from the highest education level answered by parents from 1st wave to 4th wave, and other variables adopted average values. Based on model 1-2, the family support and low perceived likeability in model 2-1 (simultaneous effects) were utilized the corresponding annual total scores. Based on model 2-1, the family support and low perceived likeability of model 2-2 (lag effects) were utilized the annual total scores in the previous year*.

† *p < 0.1*;

* *p < 0.05*;

** *p < 0.01*;

*** *p < 0.001*.

According to the time-dependent models, model 2-1 implied that high parental supervision (HR = 0.85, 95% CI = 0.74–0.99), high family support (HR = 0.91, 95% CI = 0.86–0.97) and low family conflict (HR = 1.26, 95% CI = 1.03–1.53) in the current year could protect children and adolescents from drug-use activities. In model 2-2, we considered the lag effects of social attachment factors on experimental drug use and discovered that sustained high parental supervision (HR = 0.77, 95% CI = 0.66–0.89) and low family conflict (HR = 1.23, 95% CI = 1.001–1.52) may reduce students' experimental drug use rates.

Based on the above analysis, sex as a control variable was significant in each model. Given the possible differential risk/protective effects of sex, time-dependent effects were further examined by a gender-stratified analysis. The model with simultaneous effects showed that boys who were less supported by their family were more likely to have experimental drug use, while girls who were less supervised by their parents were more likely to have experimental drug use. The results of the lag effects model showed that both boys and girls were more likely to develop experimental drug use in the next year if they had low degree of parental supervision, but the significance of the model was *p* = 0.001 for girls and *p* = 0.048 for boys.

## Discussion

Based on our 11-year prospective cohort study, the new incidences of experimental drug use among students (both annually and during each educational stage), and the time-dependent effects could be assessed. There were two peaks of students' experimental drug use: in grades 9 (i.e., students' first experience with entrance examinations) and 13 (i.e., the first-time students left their family and enjoyed life/freedom alone). Notably, experimental drug use in senior high school and university was much higher than before. Moreover, our findings revealed the importance of social attachment for preventing students from experimentally consuming drugs. It was essential to avoid family conflict/low perceived likeability during childhood and to emphasize the necessity of sustained parental supervision and sustained family support.

### Possible Reasons for Differences in Experimental Drug Use Among Students at Different Education Stages

Most research suggests that early (12–14 years old) to late (15–17 years old) adolescence is a critical risk period for the initiation of substance use (71). Some researchers indicated that adolescents first start using drugs between the ages of 13 and 17 years (72). In this study, we found that both the third year of junior high school and the first year of university were the time points when experimental drug use increased. There is excessive academic pressure in the third year of junior high school, and a few students may use drugs to escape the pressure (i.e., there may be other related reasons). A qualitative study of 38 adolescents using focus interviews found that academic pressure is a major source of stress. These adolescents attempted to relieve stress by using substances, especially when they felt that they could not succeed (73). Furthermore, with decreased parental supervision and increased autonomy, a small number of students who enter university may exhibit more participation in high-risk behaviors such as drug use (74, 75). In this study, students' drug use decreased in the first years of junior and senior high school likely because during these two periods, students are entering a new stage of learning, are adapting to the environment and do not yet feel great pressure due to learning or peer relationship distress. Hence, it suggests that we should pay more attention to the status of students at each stage to protect them from exposure to drugs.

### The Key Role of Social Attachments in Reducing Drug Use in Children and Adolescents

As is currently known, social attachments are important (including attachment relationships between family members/peers and adolescents) in shaping adolescents' behaviors. Because youths enjoy maintaining social relationships with their families and friends, the risk behaviors of children and adolescents are especially affected by or imitated from their parents and peers. For example, studies have shown that the usage of amphetamine, cocaine and cannabis among adolescents before attending university was mostly associated with illicit drug use by their parents and friends (76). Stronger social attachments may lead to the avoidance of illegal and heavy substance use (31). Some studies pointed out that the strongest protective factors against substance use among senior high school students are individual factors (such as substance use refusal self-efficacy and self-control), peer factors (such as attitudes against substance use and peer drug use), and family support (such as the ability of parents to listen) (77, 78). Among these factors, the factors related to the family environment are particularly important (79). The results of this study confirmed the importance of sustained family support/parental supervision in reducing substance use among children and adolescents. The negative impact of poor parental involvement in children's education on adolescents' substance use before they attend university should not be ignored (76). Parents should participate more in their children's studies and life before they attend university to prevent their children's hedonism, which can reduce their opportunity to access illicit drugs.

Moreover, our study revealed that low perceived likeability from peers was an influential factor of experimental drug use, especially for exposure in childhood. Those with high levels of family conflict in grade 4 were at an increased risk of experimental drug use during the follow-up, which was consistent with previous research findings (62). Regarding the measurement of family conflict, the types covered in this study included parents fighting with each other verbally, siblings fighting with each other verbally or physically, and adolescents fighting with parents or other adults in the family. Future research could further investigate this relationship by grouping family conflict. Past research has suggested that girls with low perceived likeability are more likely to develop future substance use as adults (52). Similarly, our study discovered that low perceived likeability in grade 4 was associated with an increased risk of experimental drug use during the follow-up. Therefore, high levels of family conflict and low perceived likeability are risk factors for experimental drug use in students.

### Gender Difference in Experimental Drug Use Among Students From Childhood to Adolescence

Parents may raise their children differently by gender because of the stereotype and social expectations for boys/girls. Studies have found that compared with girls, boys are more likely to engage in high-risk behaviors, such as aggressive behavior and drinking (80–82); while girls tend to show empathic behaviors and prosocial behaviors. Similarly, our study revealed that boys were more likely than girls to consume drugs. In addition, continuous parental supervision of students, including both boys and girls, from childhood to adolescence is necessary to reduce their possibility of exposure to drugs and then decrease the likelihood of experimental drug use. For boys, appropriate family support is also a key protective factor in avoiding experimental drug use. This study separated different social attachment factors for experimental drug use behaviors among boys and girls, which suggested that we should take gender-specific measures to prevent students' experimental drug use behaviors in the beginning.

While drug use may cause harm and further addiction, it is a gradual process and it is not absolute. It should be taken into account that many people who have ever consumed drugs a time may not do this again, only use occasionally or use casually without developing any significant issues. Based on the findings of the Monitoring the Future Survey (MTF) of America from 1975 to 2016, most 12th graders disapproved of the regular use of any illicit drugs, and fewer respondents uncovered disapproval of experimental or occasional use than of regular use (83), which may be related to their change trends in the perceived risk of use (84). A previous study revealed that compared to students who reported “often” drug use, those who reported “once or twice” or “sometimes” use and those who never endorsed use were more similar to each other (85), such as both reported high levels of hope for the future (86). Because the cohort study just reported children's and adolescents' experimental drug use, those who initiated the use of illicit drugs might not continue use, not necessarily become an addictive behavior. Therefore, the results of the current study should be interpreted with caution.

### Strengthens and Limitations

The main strength of this study is its long-term prospective cohort design, which allowed us to discover the annual incidence rate and mean annual incidence rate of experimental drug use from childhood to adolescence by education stage, and examine the longitudinal effects of social attachment factors on experimental drug use behavior. Particularly, this cohort study was based on a school-based survey focusing on non-clinical samples. Our findings are noteworthy. However, some limitations still exist. First, cohort studies inevitably experience a loss to follow-up over more than 10 years of visits. Fortunately, the differences in the distributions of several important variables (as previously mentioned between “the first-wave data” and “the 11-year follow-up data”) were non-significant, and there were no differences in the HRs before and after the MI procedures. Second, the questionnaire used in the present study focused on general problems faced by children and adolescents in campus and family lifestyles. The questionnaire was not specific to drug problems; thus, we were unable to obtain more covariates and provide further explanation. Third, because the questionnaire asks about children's behavior over time, recall bias could occur possibly due to incorrect long-term memory. We believe that recall bias is likely minimal since drug use is a rare behavior that is not easily forgotten. As drug use is a sensitive issue, students might not report it honestly. Therefore, reporting bias could exist. In this regard, our well-trained interviewers established good communication with the interviewees at the beginning of the questionnaire to reduce their defensiveness, informed them that their privacy would be fully protected and that the content of the questionnaire would not reflect personal information, and placed some sensitive questions at the back of the questionnaire. Fourth, this study is a school-based longitudinal follow-up study, and there may be attrition bias due to students dropping out. This limitation needs to be taken into account when interpreting the results. Finally, this study is also limited by the different measurement approaches used to assess particular items across the years. Therefore, when using time-independent covariates, if the items were not measured in 1st wave, variable values from the closest available year were used. When using time-dependent covariates, the values of the variables from the preceding year were used.

## Conclusion

Based on the findings of this study concerning the importance of social attachment, we should fully mobilize the positive forces of family, peers and school to create a healthy environment for children and adolescents and even create a positive social climate. For every dollar spent on prevention, at least ten dollars can be saved in future health, social and crime costs (87). In terms of family, according to the United Nations Office on Drugs and Crime (UNODC), parents are important because families are a primary source of socialization and parental opinion can either reinforce or countermand the messages conveyed by drug abuse prevention programmes (88). Parents should be encouraged to use a warm child-rearing style with the help of parenting skills programmes which support parents in being better parents (89). What's more, parents should take the initiative to disseminate correct health information to their children and ensure adequate parent-child communication to enhance family bonding. Regarding peers, children and adolescents are encouraged to monitor and support each other through mutual help groups to maximize the positive influence of peers in their social network. Regarding school, drug-related learning outcomes should be addressed in the context of the health curriculum or other appropriate learning areas (90). Starting with the ongoing comprehensive and developmental elements that encourage the development of personal and social skills and values, the designed curriculum should cover adolescent development, stress coping, sexuality, and collaboration between home and school and personal relationships.

In summary, the risk of experimental drug use increases with age but may decrease due to protective factors. Additional prevention resources for young people exposed to multiple contextual risk factors (even in the absence of risk behaviors) from childhood to adolescence were offered by Wang et al. (91). Hence, preventative strategies should be implemented at early stages to avoid the onset of drug-use behaviors among students. Firstly, we recommend that parents expand efforts to lower conflict among families or relatives and provide children with sustained support and behavioral supervision. It is also necessary to understand their child's acceptance from others, to provide emotional support and effective communication skills when their child experiences difficulties. Secondly, teachers should focus on the social attachment status of their students while considering their attachments to their families and peers. Particularly, it is crucial to launch continuous and cross-stage drug-use denial campaigns aiming to prevent drugs from entering school campuses. Thirdly, relevant government departments should formulate corresponding policies, such as advocating health education courses or activities becoming compulsory credits, establishing a support association or counseling services aiming to sustain increases in students' drug refusal efficacy as well as health consciousness, and nurturing conflict resolution and stress management skills in children and families. Finally, although the first attempt at drugs does not mean that they will become dependent or drug abusers in the future, as far as the prevention of drug use by children and adolescents is concerned, it is still necessary to avoid the opportunity of the first try. Stigma is not as important as providing supportive environment to help children who want to get out of drug use.

## Data Availability Statement

The datasets used and/or analyzed during the current study are available from the corresponding author on reasonable request.

## Ethics Statement

The studies involving human participants were reviewed and approved by Institutional Review Board in National Health Research Institutes in Taiwan (No. EC9009003). Written informed consent to participate in this study was provided by the participants' legal guardian/next of kin.

## Author Contributions

Y-CC, XL, C-CW, and H-YC were major contributors in conception and design of the study. Y-CC, C-CW, and H-YC participated in the data collection. Y-CC, XL, C-YL, SZ, and H-YC made contributions to data analysis and interpretation. Y-CC, XL, and C-YL drafted the manuscript. SZ, H-YC, and Y-CC supervised the study and critically reviewed the manuscript several times. Y-CC provides funding acquisition. All authors agree to be accountable for all aspects of the work in ensuring that questions related to the accuracy or integrity of any part of the work are appropriately investigated and resolved and read and approved the final manuscript.

## Funding

This article was funded by the Scientific Research Grant of Fujian Province of China (No. Z0230104). The sponsors of the project had no role in the study design, data collection, data analysis, data interpretation and in writing the manuscript.

## Conflict of Interest

The authors declare that the research was conducted in the absence of any commercial or financial relationships that could be construed as a potential conflict of interest.

## Publisher's Note

All claims expressed in this article are solely those of the authors and do not necessarily represent those of their affiliated organizations, or those of the publisher, the editors and the reviewers. Any product that may be evaluated in this article, or claim that may be made by its manufacturer, is not guaranteed or endorsed by the publisher.
