# Supplementary material for: Effects of Social Attachment on Experimental Drug Use From Childhood to Adolescence: An 11-Year Prospective Cohort Study
Source: Front Public Health. 2022 Mar 29;10:818894. doi: 10.3389/fpubh.2022.818894 (PMC9002116; doi:10.3389/fpubh.2022.818894)
Supplement: Supplementary file 1 [file Table_1.DOCX]

**APPENDIX 1 |** Schoenfeld test for Proportional Hazards Assumption

| Variables | Maximum Absolute Value | Replications | Seed | *p* |
| --- | --- | --- | --- | --- |
| Subjective academic performance | 0.52 | 1000 | 31418285 | 0.58 |
| Parent’s education level | 0.99 | 1000 | 31418285 | 0.11 |
| Parental supervision | 0.90 | 1000 | 31418285 | 0.29 |
| Family support | 0.86 | 1000 | 31418285 | 0.26 |
| Family conflict | 0.51 | 1000 | 31418285 | 0.66 |
| Low perceived likeability | 0.47 | 1000 | 31418285 | 0.68 |
